# Anuerismal Obstruction of Vena Cava Superior with Special Reference to the Caval Syndrome

**Published:** 1917-06

**Authors:** 


### Abstracts and Selections.

### Anuerismal Obstruction of Vena Cava Superior With Special Reference to the Caval Syndrome.

Skillern reports in the *International Clinics* an example of this condition and also gives a brief review of the literature. The caval syndrome is described as follows:

This consists of enormous edematous swelling of the head, neck, trunk, upper extremities, and marked obstruction of the veins. These clinical manifestations depend upon the formation of a collateral circulation, the extent of narrowing of the vena, and the size and extent of the pathologic process which causes the compression.

The first result of compression is obstruction of the venous blood in the entire territory of the vena cava superior. Through dilatation of all veins and capillaries in the territory of the upper half of the body an enormous cyanosis is often caused. The result of the obstructed outflow of venous blood while more blood is continually being brought to the part is the appearance of edema. From the distribution of the edema and its further advance one may draw diagnostic conclusions as to the site of compression. The lower half of the body is almost always free from edema, but the latter appears here as well, when through overdistension of the inferior vena cava obstruction in the tributaries of this vein results, or when through cardiac weakness edema appears in the lower extremities and scrotum. Usually, however, even in this case the swelling of the upper half of the body remains in characteristic contrast to the very much slighter edema of the lower. Not only the subcutaneous cellular tissue, but also the deeper parts are involved by the edema, especially the mediastinum. Of importance also is edematous infiltration of the mucous membranes, for thus edema of the glottis may give ground for suddenly appearing death.

In the *diagnosis* of compression of the superior vena cava but little difficulty is encountered. The diagnosis is based upon the obstructive signs appearing in the territory of the vena cava superior; i. e., upon the direction of a collateral circulation and the prominence and characteristic course of the veins belonging to it. In favor of aneurism as the cause are the appearance of a dull, pulsating area and the Oliver-Cardarelli symptom.—*International Clinics.*

